# Metacaspase gene family in Rosaceae genomes: Comparative genomic analysis and their expression during pear pollen tube and fruit development

**DOI:** 10.1371/journal.pone.0211635

**Published:** 2019-02-22

**Authors:** Yunpeng Cao, Dandan Meng, Tianzhe Chen, Yu Chen, Wei Zeng, Lei Zhang, Qi Wang, Wei Hen, Muhammad Abdullah, Qing Jin, Yi Lin, Yongping Cai

**Affiliations:** 1 School of Life Sciences, Anhui Agricultural University, Hefei, China; 2 College of Horticulture, Anhui Agricultural University, Hefei, China; ICAR-National Research Centre on Plant Biotechnology, INDIA

## Abstract

Metacaspase (MC), which is discovered gene family with distant caspase homologs in plants, fungi, and protozoa, may be involved in programmed cell death (PCD) processes during plant development and respond abiotic and biotic stresses. To reveal the evolutionary relationship of *MC* gene family in Rosaceae genomes, we identified 8, 7, 8, 12, 12, and 23 *MC* genes in the genomes of *Fragaria vesca*, *Prunus mume*, *Prunus persica*, *Pyrus communis*, *Pyrus bretschneideri* and *Malus domestica*, respectively. Phylogenetic analysis suggested that the *MC* genes could be grouped into three clades: Type I*, Type I and Type II, which was supported by gene structure and conserved motif analysis. Microsynteny analysis revealed that *MC* genes present in the corresponding syntenic blocks of *P*. *communis*, *P*. *bretschneideri* and *M*. *domestica*, and further suggested that large-scale duplication events play an important role in the expansion of *MC* gene family members in these three genomes than other Rosaceae plants (*F*. *vesca*, *P*. *mume* and *P*. *persica*). RNA-seq data showed the specific expression patterns of *PbMC* genes in response to drought stress. The expression analysis of *MC* genes demonstrated that *PbMC01* and *PbMC03* were able to be detected in all four pear pollen tubes and seven fruit development stages. The current study highlighted the evolutionary relationship and duplication of the *MC* gene family in these six Rosaceae genomes and provided appropriate candidate genes for further studies in *P*. *bretschneideri*.

## Introduction

Programmed cell death (PCD) is a developmental and genetically controlled cell death process, which is divided into two broad categories: environmentally induced PCD and developmentally regulated PCD in plants [1–4]. Environmentally induced PCD is primarily caused by external abiotic or biotic signals, such as drought, hormone, heat shock and pathogens stresses [5–8]. In contrast, developmentally regulated PCD covers most of the organs and tissues of plants, such as fruit, root, stem, leave and xylem, which caused by internal factors and occurs at predictable locations and times [9–11].

Metacaspases (MCs) are multifunctional proteins that are involved in PCD regulation, cell cycle and senescence, and oxidative stress. According to the sequence similarities and domain structure, plant MCs are divided into two types: Type-I and Type-II [1, 12]. Additionally, a subgroup appeared from the Type-I and named as Type-I*, which exclude N-terminal zinc-finger motif [1]. These different types MC proteins contained a putative conserved caspase-like domain (PF00656), which consists of p10 and p20 subunits [1, 12]. Additionally, Type-I MC proteins have a short linker between the p20 and p10 subunits and an N-terminal pro-domain upstream of the p20 subunit. Type-II MC proteins did not contain N-terminal pro-domain upstream of the p20 subunit, but it has a longer linker between the p20 and p10 subunits than Type-I MC proteins [1, 12, 13].

*MC* genes play an important role in stress-induced and developmentally regulated PCD. Tsiatsiani et al. (2011) have been identified nine *MCs* (*AtMC1*−*AtMC9)* in *Arabidopsis thaliana* [2]. Under the stress of UV-C and H_2_O_2_, the expression of *AtMC8* was up-regulated in *A*. *thaliana*, which accelerated the process of the PCD in protoplasts [14]. *AtMC1* and *AtMC2* have been reported as positive and negative regulators, respectively, to antagonize pathogen-triggered PCD [15]. *AtMCP2b* (i.e. *AtMC5*) can activate apoptotic-like cell death during early senescence process and oxidative stress [16]. *AtMC9* plays an important role in the process of autolysis during vessel cell death [9]. Additionally, the *Nicotiana benthamiana NbMCA1*, *Capsicum annuum CaMC9*, *Triticum aestivum TaMC4* have been reported to function in stress response and PCD [4, 6, 17].

Systematic analysis and genome-wide identification of *MC* gene family have been reported in *Hordeum vulgare*, *Solanum lycopersicum*, *Hevea brasiliensis*, *Vitis vinifera*, *Arabidopsis thaliana* and *Oryza sativa* [18–23]. However, investigations on the *MC* gene family in Rosaceae genomes are limited. *F*. *vesca*, *P*. *mume*, *P*. *persica*, *P*. *communis*, *P*. *bretschneideri* and *M*. *domestica* are important worldwide cultivated fruit trees, and they belong to Rosaceae family [24–29]. In the present study, we identified *MC* gene family members in these six genomes by screening these genome sequences using bioinformatics approaches. Then we characterized their phylogenetic relationship, gene structures, chromosomal distribution, microsynteny and expression patterns. The current study explored the evolutionary relationship of *MC* gene family members in the Rosaceae plants and provided insights into the functions of *PbMCs* during *P*. *bretschneideri* pollen tube and fruit development.

## Materials and methods

### Identification and characterization of the *MC* gene family in Rosaceae genomes

Genome resources and predicted proteins of *P*. *bretschneideri* (version 1.0) was downloaded from the GigaDB (http://gigadb.org/), *P*. *persica* (version 1.0) from Ensembl Plants (http://plants.ensembl.org/index.html), *P*. *mume* (version 1.0) from PGDD database (http://chibba.agtec.uga.edu/duplication/), *F*. *vesca* (version 2.0) from PLAZA v2.5 (https://bioinformatics.psb.ugent.be/plaza/versions/plaza_v2_5/), *P*. *communi* (version 1.0) from Pear Genome Project (http://peargenome.njau.edu.cn/), *M*. *domestica* (version 3.0) from GDR database (https://www.rosaceae.org/). Three strategies were used to identify gene-encoding MC from six Rosaceae plants at the whole-genome level. First, the published *Arabidopsis thaliana* MC protein sequences were used to query putative MC homologous proteins of *F*. *vesca*, *P*. *mume*, *P*. *persica*, *P*. *communis*, *P*. *bretschneideri* and *M*. *domestica* using BLASTp software with E-value cutoff 1×10^−5^. Subsequently, HMM (Hidden Markov Model) profiles of the Caspase-like domain (PF00656) in the Pfam database was searched against the local database using HMMER 3.0 software with E-values cutoff 1×10^−3^ [30, 31]. Finally, the Pfam and Smart databases were used to check these candidate sequences that contained Caspase-like domain (PF00656) [32, 33]. Additionally, we used the MEROPS online tool (https://www.ebi.ac.uk/merops/) [2] to predict the distribution of Type-I*, Type-I and Type-II *MC* genes in these Rosaceae genomes.

### Sequence alignment and phylogenetic analysis of MC proteins in Rosaceae genomes

To further insight into the phylogenetic relationship of MC proteins in *F*. *vesca*, *P*. *mume*, *P*. *persica*, *P*. *communis*, *P*. *bretschneideri* and *M*. *domestica*, we aligned multiple sequence alignments including FvMCs, PmMCs, PpMCs, PcMCs, PbMCs and MdMCs using MAFFT software [34, 35]. For all MC proteins, we determined the best substitution model using modeltest software [36]. Subsequently, th**e** IQ-TREE software was used to generate the Maximum Likelihood (ML) tree with 1000 bootstrap replications and VT+G4 model [37].

### Gene structures and chromosomal locations of *MC* genes in Rosaceae genomes

To determine the location of *MC* genes on chromosomes, the published *Arabidopsis thaliana MC* genes were used as query sequences against local Rosaceae genome database using BLAST software [38]. To display gene structure of each *MC* gene, the GSDS website was used to parse GFF3 files and visualize them [39]. The MEME program Version 4.11.1 was used to identify the conserved motifs according to previously published manuscripts [40–42].

### Microsynteny analysis of *MC* genes in Rosaceae genomes

The microsynteny analysis of each *MC* gene was carried out using Microsyn software and MCScanX pipeline [43, 44], based on the previous description [39, 45, 46]. Firstly, three files (i.e. the gene identifier file, the CDS file and the gene list file) were generated. Subsequently, we used local BLAST software to compare whole proteins of each species with E-value less than 1e^−10^. The position and blast output files of all protein-coding genes were imported into MCScanX software to scan the collinearity gene pairs and the Circos software was used to display the results of collinearity gene pairs [47]. Nonsynonymous (*Ka*), synonymous (*Ks*) and *Ka*/*Ks* ratios were estimated using DnaSP v5 software [48].

### Expression analysis of *MC* genes

In the present study, the RNA-Seq data were downloaded from the public NCBI database, and then these data was used to survey the expression of *MC* genes. The accession numbers and sample details for above the RNA-Seq data have mentioned in the availability of data and materials section. The low-quality base-calls (Q < 20) of raw reads were deleted by FASTX-toolkit. The clean reads were mapped to the reference genome using TopHat2 software with default parameters [49, 50]. Finally, we used the Cufflinks software to assemble and estimate the expression FPKM values [50, 51]. The R software was used to visualize the gene expression profiles of *MC* genes.

### Quantitative real‑time PCR (qRT-PCR)

The methods for collection and drying, and in *vitro* culture of pear pollen grains were based on the procedures according to the previously published manuscripts [52, 53]. The pollen samples were collected from 40-year-old pear trees (*Pyrus bretschneideri*. Rehd), and mature pollen was immediately frozen in liquid nitrogen and stored at −80°C until use. The pollen grains were cultured in this medium for hydration, germination and growth. The medium components were 450 mM sucrose, 25 mM 2-(N-morpholino) ethanesulfonic acid hydrate, 15% (w/v) PEG4000, 1.5 mM H_3_BO_3_ and 0.5 mM Ca(NO_3_)_2_, with pH 6.0–6.5 (pH was adjusted with KOH). The guanidine thiocyanate extraction method was used to isolate the total RNAs by RNA Plus (Takara) [54, 55]. According to the manufacturer’s instructions, first-strand cDNAs were synthesized from DNaseI-treated total RNA using Oligo(dT) primers and reverse transcriptase (TIANGEN, China). The qRT-PCR was conducted on a CFX96 Touch Real-Time PCR Detection System (BIO-RAD), based on the manufacturer’s protocol. Primer Express 3.0 software (Applied Biosystems) was used to design the primers of *PbMC* genes for qRT-PCR (S1 Table). Relative expression levels of *PbMC* genes were normalized against the *P*. *bretschneideri* Actin gene (NCBI ID AF386514). The relative expression level was calculated as 2^−ΔΔCt^ as described previously [41, 56]. In the present study, three biological replicates were conducted for each sample.

## Results

### Identification of *MC* genes in Rosaceae genomes

To identify the *MC* gene family members in Rosaceae genomes, HMM model and BlastP were used to search against the whole-genome sequence, with the procedures described previously [45, 57]. We identified 70 genes as members of *MC* gene family in these six Rosaceae genomes, including 8 *FvMC* genes in *Fragaria vesca*, 7 *PmMC* genes in *Prunus mume*, 8 *PpMC* genes in *Prunus persica*, 12 *PcMC* genes in *Pyrus communis*, 12 *PbMC* genes in *Pyrus bretschneideri* and 23 *MdMC* genes in *Malus domestica*. The identified *MC* genes were designated as *FvMC*, *PmMC*, *PpMC*, *PcMC*, *PbMC*, and *MdMC* followed by number. The theoretical *pI* values of 24 MC proteins, such as MdMC08 (9.13), FvMC06 (8.98) and FvMC06 (8.53) were above 7, suggesting that these proteins were alkaline, however, the remaining sequences encoded by the other *MC* genes were acidic (<7). The detailed parameters of *MC* genes were listed in S2 Table, including theoretical *pI*, molecular weight, protein length, and chromosome location.

### Phylogenetic and structural analysis of *MC* genes in Rosaceae genomes

**Fig 1:**
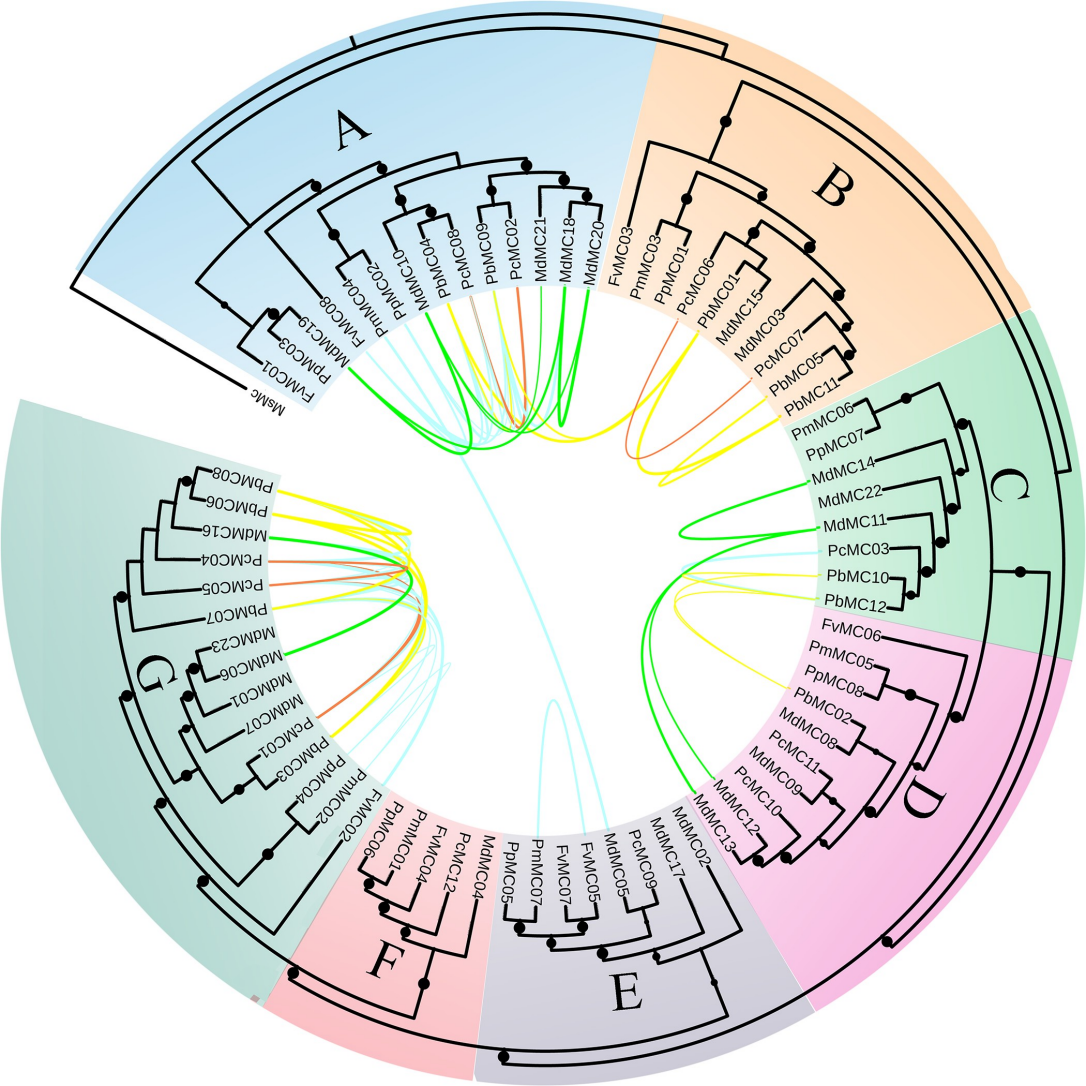
Phylogenetic tree of *MC* genes from six Rosaceae species, including *F*. *vesca*, *P*. *mume*, *P*. *persica*, *P*. *communis*, *P*. *bretschneideri* and *M*. *domestica*. The Maximum-likelihood tree was generated using IQ-TREE software, and *MsMC* (CBN76943.1) from *Ectocarpus siliculosus* was used as the out-group. The size of the point corresponds to the size of the bootstrap value. The yellow, red and green lines highlight the paralogous gene pairs of *MC* from the *P*. *bretschneideri*, *P*. *communis*, *M*. *domestica*, respectively. However, the blue lines highlight the orthologous gene pairs of *MC* among these six Rosaceae genomes.

MCs could be grouped into three classes (Type I*, Type I and Type II) according their conserved domains [2]. In the present study, to obtain insight into the phylogenetic relationships and evolutionary history in the MC family, MC proteins from *F*. *vesca*, *P*. *mume*, *P*. *persica*, *P*. *communis*, *P*. *bretschneideri* and *M*. *domestica* were compared for comprehensive phylogenetic analysis. As shown in Fig 1 and S1 Fig, the Maximum-likelihood tree of these *MC* genes was classified into three classes, which was supported the above discrimination based on their conserved domains. Subsequently, we found that the class I contained 42 type I *MC* genes, and it was further divided into four subclasses: A, B, C and D with each subclass having 14, 10, 8 and 10 members, respectively. The class II had 20 type II *MC* genes, and it was categorized into two subclasses: F and G, with each class containing 5 and 15 members, respectively. The class III contained 8 type I* *MC* genes, which is named E. *MC* genes in *P*. *mume*, *P*. *persica*, *P*. *communis* and *M*. *domestica* distributed in all the seven classes, but there are no members from *F*. *vesca* in class C and no members from *P*. *bretschneideri* in class E and F (Fig 1).

**Fig 2:**
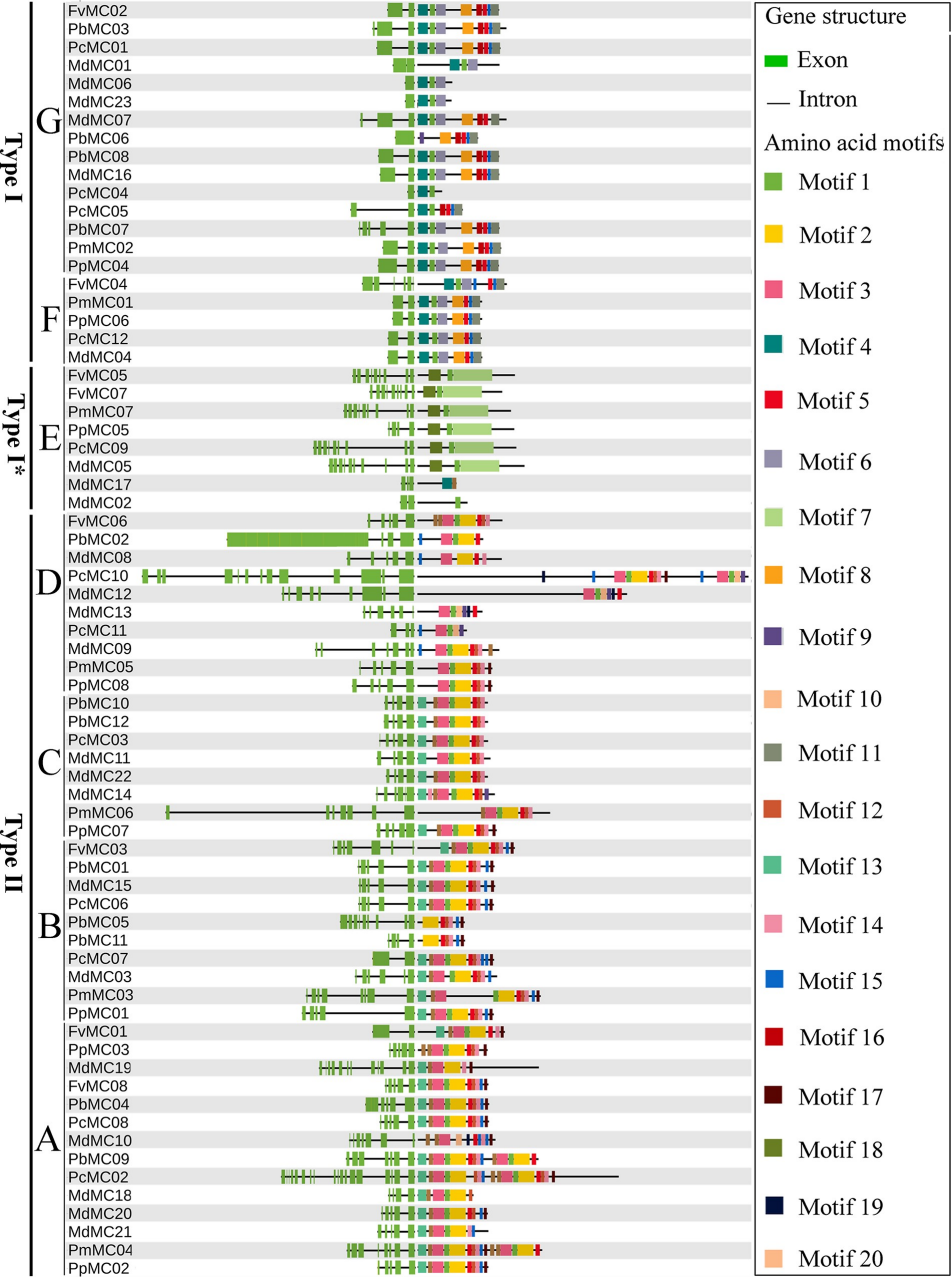
Exon-intron structure and conserved protein motif analyses of *MC* genes from six Rosaceae species. Exons and introns are indicated by green and grey, respectively. All motifs were scanned by MEME online tool using the complete amino acid sequences of all MC proteins from six Rosaceae species.

To gain understanding in the structural diversity of *MC* gene family members, we built exon-intron organization maps of each *MC* gene, as shown in Fig 2. The 70 *MC* gene family members have different numbers of introns, ranging from 1 to 17, with the exception of *MdMC06*, *MdMC23*, *PbMC06*, *PcMC06* and *PcMC04* having no introns. While *PcMC02* containing the highest introns (17) among these *MC* genes. Additionally, we found that the most *MC* gene family members clustered in the same subfamily contained similar exon-intron (i.e. intron numbers and exon length) distribution patterns. For example, four genes (*PmMC01*, *PpMC06*, *PcMC12* and *MdMC04*) locating in the subfamily F shared one intron, and four genes (*FvMC06*, *MdMC08*, *PmMC05* and *PpMC08*) belonging to subfamily D had four introns. Likewise, most *MC* gene family members in the subfamily A had no or one intron except *PbMC03*, *MdMC07* and *PbMC07*, which contained 2, 2 and 4 introns, respectively. Subsequently, we also investigated the conserved motifs of MC proteins from Rosaceae genomes to understand the diversified functions of these proteins. Twenty conserved motifs were identified and designated as motif 1 to motif 20 in MC proteins, as shown in S3 Table and Fig 2. Additionally, we used the Pfam and SMART database to annotate each of the putative motifs from MEME software. Motif 1, motif 2, motif 3, motif 4, motif 6 and motif 10 were found to encode the Caspase-like domain (PF00656) and were scanned one or more times among all the seven subfamily members. For example, motif 1 and motif 3 were scanned in subfamily F, and motif 1, motif 2, motif 3 and motif 10 were scanned in subfamily C. Remarkably, most MC members within the same subfamily, especially the most closely related members (e.g. PmMC05/PpMC08, PbMC01/MdMC16, PcMC05/PcMC09 and PbMC04/PcMC08), generally contained highly similar motif compositions and distributions, indicating function may be similarities among these MC proteins. We also noted that different subfamilies had great difference within motif compositions and distributions, such as proteins in subfamily C contained motif 8, motif 12, motif 13, motif 14, motif 16 and motif 17, while subfamily E members possessed motif 1, motif 7 and motif 18. At the same time, several motifs were exclusively identified in a particular subfamily, indicating that these motifs may play an important role in the subfamily, for example, motif 7 was unique to the proteins in subfamily E.

### Chromosomal distribution and intraspecies microsynteny of *MC* genes in Rosaceae genomes

**Fig 3:**
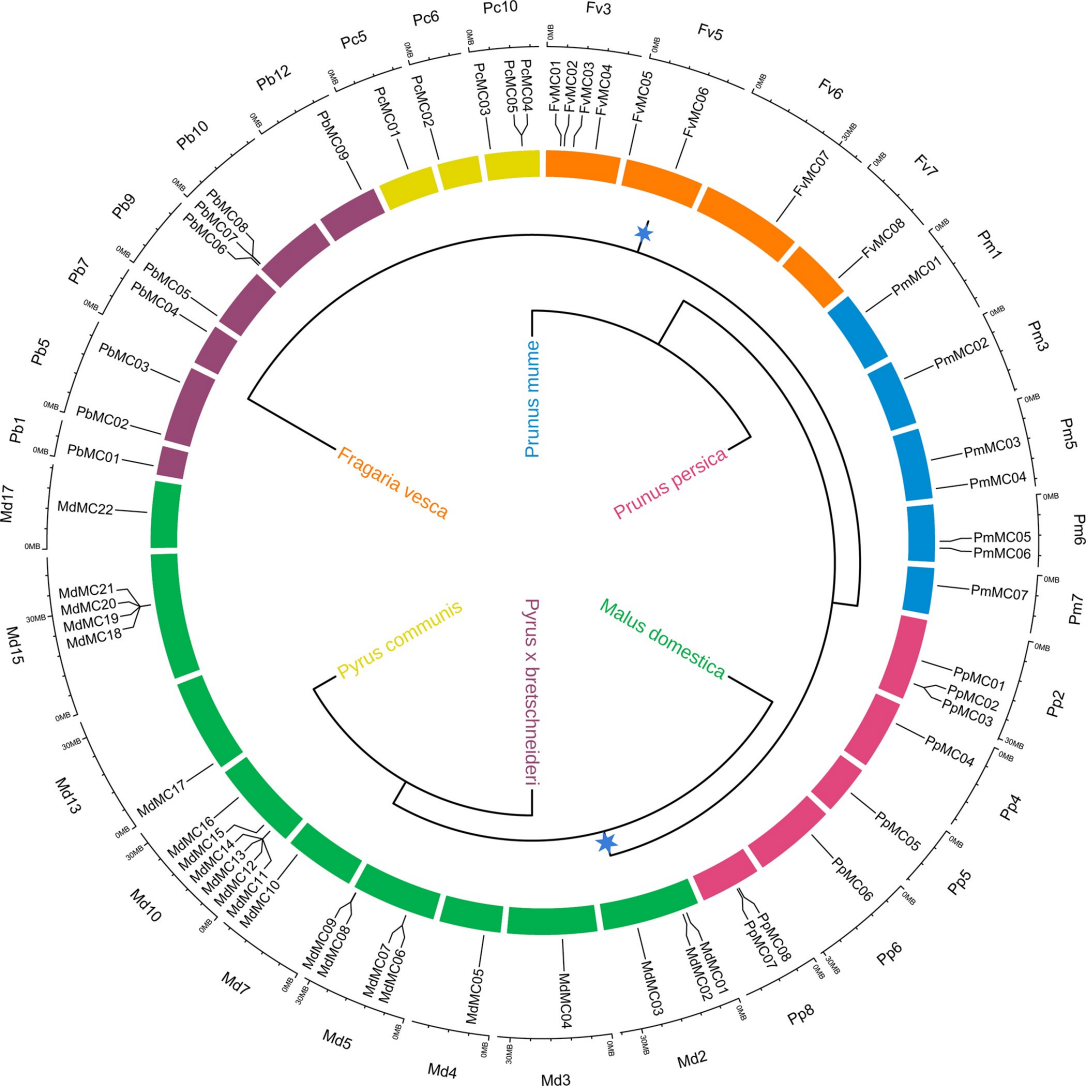
Chromosomal location of *MC* genes among *F*. *vesca* (Fv), *P*. *mume* (Pm), *P*. *persica* (Pp), *P*. *communis* (Pc), *P*. *bretschneideri* (Pb) and *M*. *domestica* (Md). Based on the GFF3 annotation information, the physical location of each *MC* was mapped. The scale is indicated by mega bases (Mb). The inner circle represents the species evolution tree, and the blue star indicated the whole genome duplication (WGD) event.

According to the positions of *MC* genes in Rosaceae genomes, we determined the physical locations of these genes among the chromosomes. These data suggested that the distribution of 70 *MC* genes on the chromosomes of the six Rosaceae species was not evenly distributed (Fig 3). For example, *FvMCs* were distributed on four of 7 chromosomes (chr 3, chr 5, chr 6 and chr 7), *PbMCs* were dispersed on six of 17 chromosomes (chr 1, chr 5, chr 7, chr 9, chr 10 and chr 12), while *PcMCs* only were dispersed on three of 17 chromosomes (chr 5, chr 6 and chr 10). For *M*. *domestica*, chromosome 10 harbors 6 *MdMCs*, the highest number among all *M*. *domestica* chromosomes (S2 Table), and followed by chromosome 15 contained 4 *MdMCs*. For *P*. *communis* and *P*. *bretschneideri*, chromosome 10 also harbors the highest number (3) among all *P*. *communis* and *P*. *bretschneideri* chromosomes (Fig 3 and S2 Table).

**Fig 4:**
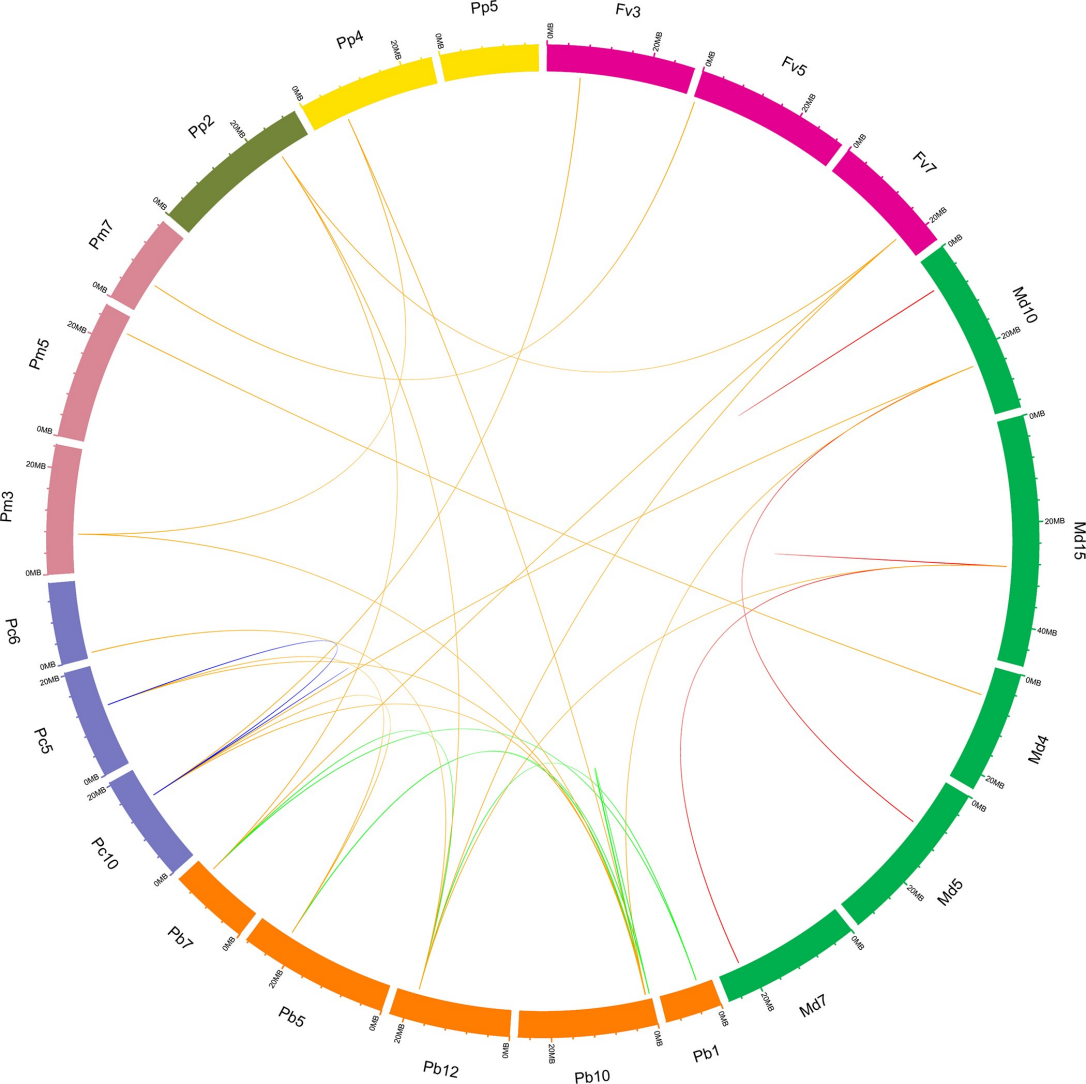
Synteny of six Rosaceae plants *MC* genes. All the syntenic MC genes were located in the map and then links by different lines. Chromosome box numbers represent sequence lengths in megabases (Mb).

**Fig 5:**
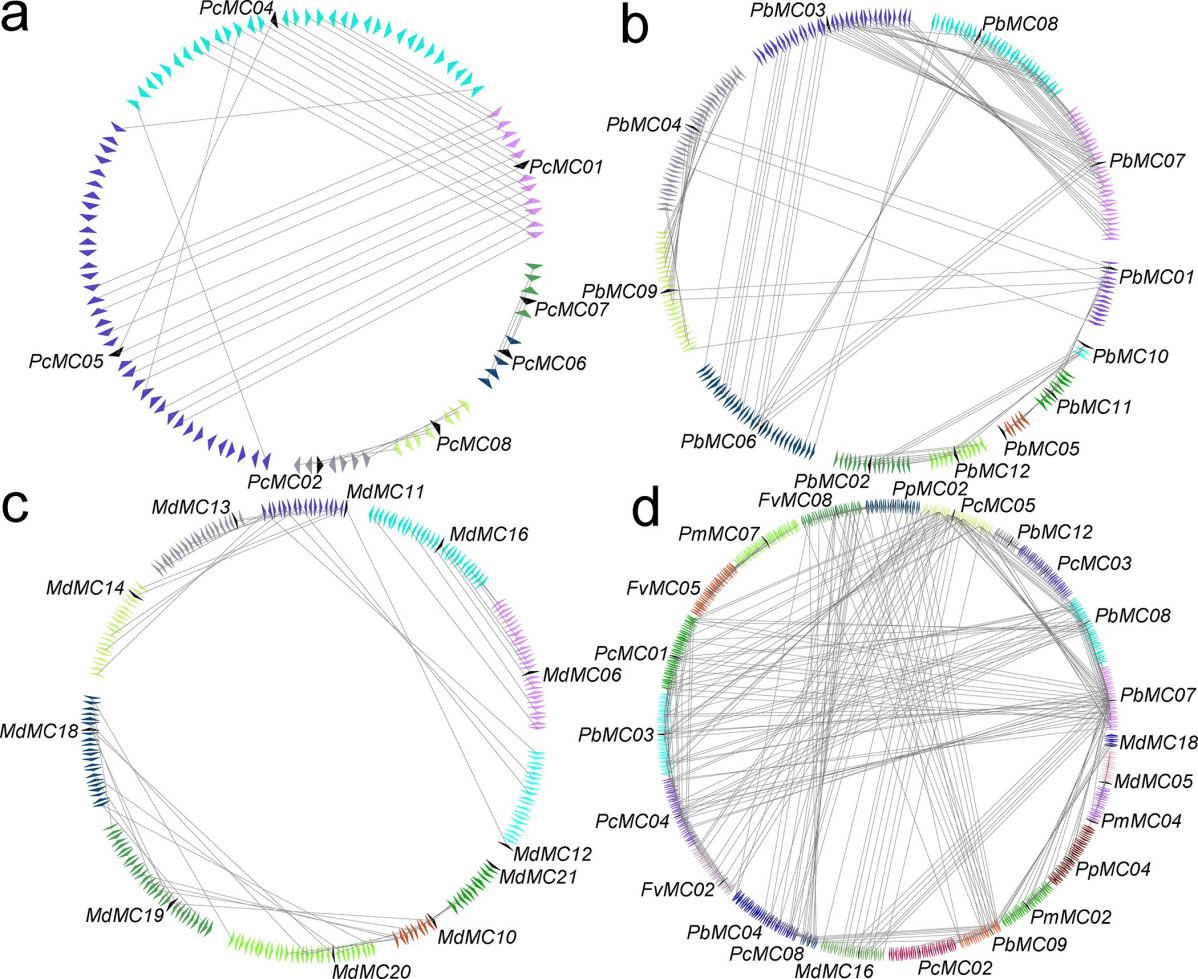
Microsynteny of *MC* gene families in *P*. *communis* (a), *P*. *bretschneideri* (b) and *M*. *domestica* (c). The Fig 5 (c) indicated interspecies microsynteny of *MC* genes in Rosaceae genomes. Genomic fragments are suggested by numbers of triangles. Black triangle represented *MC* genes, and same color indicated these genes in the same fragment.

Based on the whole genome analysis in these six Rosaceae genomes, *P*. *bretschneideri* possessed the most putative duplicated gene pairs, such as *PbMC07*/*PbMC08* and *PbMC03*/*PbMC07*, *M*. *domestica* contained nine gene pairs, *P*. *communis* had five gene pairs (S4 Table), whereas other three species not contained any gene pairs (Fig 4). It might because these *P*. *bretschneideri*, *P*. *communis* and *M*. *domestica* have experienced two genome-wide duplication events [27, 46]. Remarkably, we found that no any gene pairs were generated by tandem duplication in *M*. *domestica*, *P*. *bretschneider* and *P*. *communis*, indicating that tandem duplication may have made little or no contribution to the expansion of the *MC* gene family in these six Rosaceae genomes. Additionally, the relationships of the flanking sequences of these *MC* gene pairs were further analyzed using MCScanX and MicroSyn software (Figs 4 and 5). In *P*. *bretschneideri*, we identified three conserved genes flanking six pairs. Eight other pairs of *MC* genes had more than three pairs of conserved flanking genes, such as *PbMC07*/*PbMC08* and *PbMC03*/*PbMC07* had 17 and 16 gene pairs of conserved flanking genes, respectively. In *P*. *communis*, we found eleven conserved genes flanking two pairs, *PcMC01*/ *PcMC04* and *PcMC01*/ *PcMC05*. Three other pairs of *MC* genes (*PcMC02*/ *PcMC08*, *PcMC04*/ *PcMC05* and *PcMC06*/ *PcMC07*) contained less than five pairs of conserved flanking genes. In *M*. *domestica*, all pairs of *MC* genes had more than four pairs of conserved flanking genes, such as *MdMC06*/ *MdMC16* had eight gene pairs of conserved flanking genes. Therefore, we speculated that large-scale duplication events may contribute to the expansion of *MC* gene family members during evolution in these three Rosaceae species (Fig 5).

### Interspecies microsynteny of *MC* genes in Rosaceae genomes

For better understand the evolutionary relationship of *MC* genes among *F*. *vesca*, *P*. *mume*, *P*. *persica*, *P*. *communis*, *P*. *bretschneideri* and *M*. *domestica*, interspecies microsynteny analysis was carried out to identify orthologous *MC* genes. The flanking *MC* genes were analyzed to identify involved duplication events. In the present study, 23 out of 70 intraspecies *MC* genes were identified to involve in the interspecies microsynteny (Fig 5D and S4 Table). Among them, there were 13 orthologous gene pairs between *P*. *communis* and *P*. *bretschneideri*, 3 orthologous gene pairs between *P*. *persica* and *P*. *bretschneideri*, 2 orthologous gene pairs between *F*. *vesca* and *P*. *bretschneideri*, 2 orthologous gene pairs between *P*. *bretschneideri* and *M*. *domestica*, 1 orthologous gene pair between *P*. *mume* and *P*. *bretschneideri*, 3 orthologous gene pairs between *P*. *communis* and *F*. *vesca*, 1 orthologous gene pair between *M*. *domestica* and *P*. *communis*, 1 gene pair between *P*. *mume* and *P*. *persica*, 1 orthologous gene pair between *P*. *mume* and *F*. *vesca*, 1 orthologous gene pair between *P*. *mume* and *M*. *domestica*, and 1 orthologous gene pair between *F*. *vesca* and *P*. *persica* were identified (Fig 5D and S4 Table). Among *P*. *bretschneideri* and other five Rosaceae plants, we identified the different number of orthologous gene pairs, which may be due to different loss rates of duplicated genes during evolution.

### Expression patterns of *PbMCs* in response to drought stress

**Fig 6:**
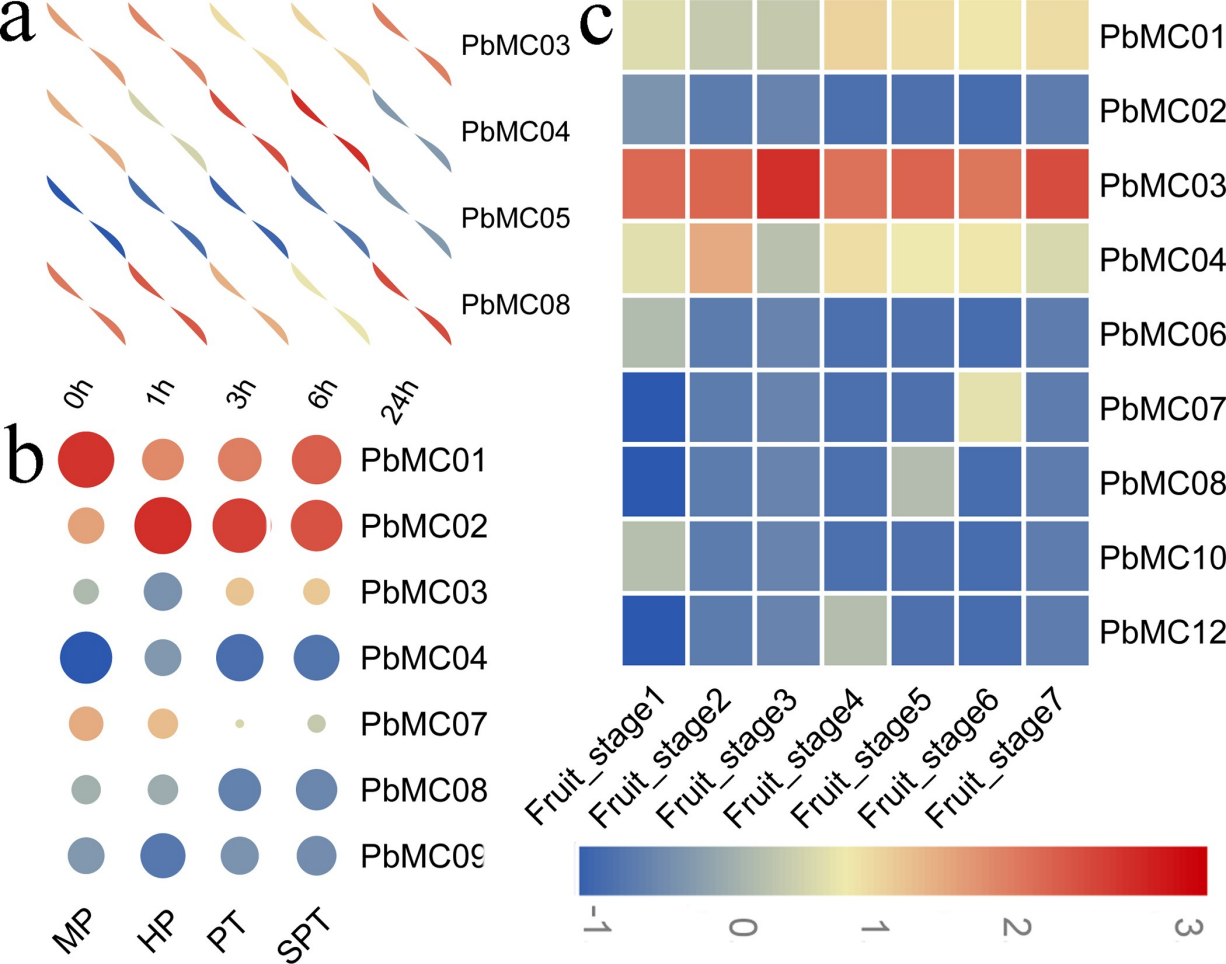
Expression analysis of *PbMC* genes under drought stress treatment (a), and/or during pear pollen tube (b) and fruit development (c). MP, HP, PT, SPT, Fruit_stage1, Fruit_stage2, Fruit_stage3, Fruit_stage4, Fruit_stage5, Fruit_stage6 and Fruit_stage7 suggested mature pollen grains of pear, hydrated pollen grains, pollen tube, top-growth pollen tube, 15 days after full blooming (DAB), 30 DAB, 55 DAB, 85 DAB, 115 DAB, mature stage and fruit senescence stage, respectively.

Drought is a major abiotic stress that can affect plant productivity, growth and development. *MCs* play an important role in the process of PCD during stress responses in plants. To further understand the possible involvement of *PbMCs* in response to drought stress, the expression profiles of *PbMCs* were analyzed by RNA-seq data. As shown in Fig 6A, only four out of 12 *PbMCs* were expressed under drought stress. Among these *PbMC* genes, three genes (*PbMC03*, *PbMC04* and *PbMC05*) displayed up-regulation at least one time point after treatment. At the same time, we also found that the expression of *PbMC08* showed continuous decrease after a long period of stress treatment, and resulting in 5-fold decrease at 6 h after treatment, and then the expression level increased after 24 h. In contrast, the *PbMC04* gene was strongly and rapidly induced by drought treatment, reached the maximum level at 6 h after treatment, suggesting the existence of a possible feedback regulatory mechanism (Fig 6A).

### Expression patterns of *PbMCs* during pollen tube and fruit development stages

**Fig 7:**
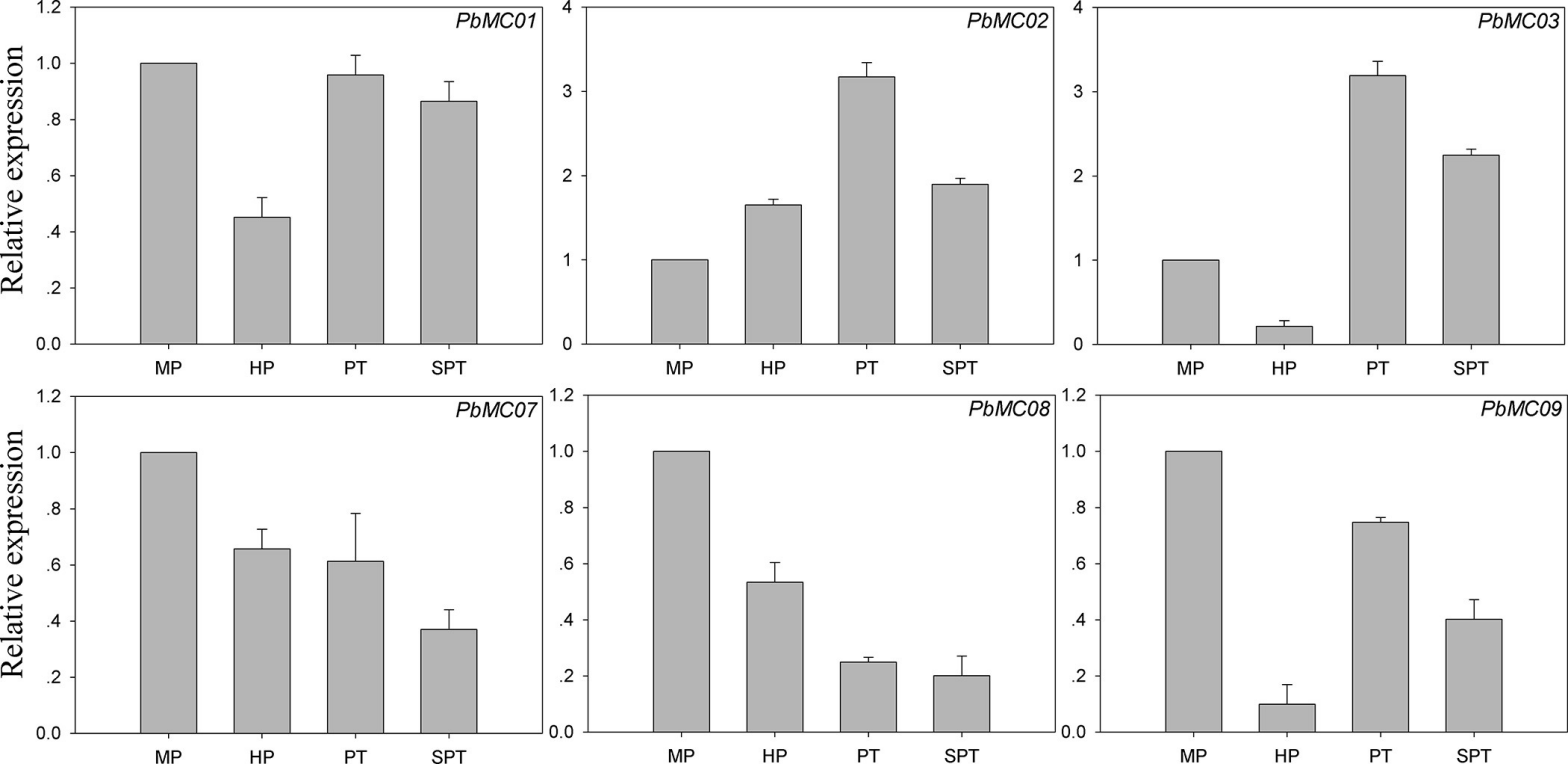
qRT-PCR analyses of *PbMC* genes during pear pollen tube development. MP, HP, PT and SPT suggested mature pollen grains of pear, hydrated pollen grains, pollen tube and top-growth pollen tube, respectively.

For flowering plants, pollen germination and pollen tube growth are very important for sexual reproduction. Tip-growth is the most important feature of pear pollen germination and pollen tube growth. The pollen tube extends very rapidly and pollen tube death occurs within 24 h of in *vitro* culture [52, 53]. These results suggested that pollen and pollen tube is an ideal model system for studying the molecular mechanisms of a series of cellular processes, such as cell growth and death. In the present study, seven *MC* genes were found transcriptionally active in at least one stage, and five genes (*PbMC01*, *PbMC02*, *PbMC0*3, *PbMC07* and *PbMC08*) out of the seven genes were identified transcriptionally active in all four stages: including mature pollen grains of pear (MP), hydrated pollen grains (HP), pollen tube (PT) and top-growth pollen tube (SPT), indicating that these genes may play a role in reproduction (Fig 6B). Subsequently, we used the qRT-PCR experiment to verify the expression patterns, and the results were shown in Fig 7. These data suggest that these *PbMC* genes showed similar expression patterns as transcriptome data.

To gain insight into the expression patterns of *PbMCs* in *P*. *bretschneideri* fruit development, gene expression profile analysis was carried out in seven pear fruit developmental stages (i.e. 15 days after full blooming (DAB), 30 DAB, 55 DAB, 85 DAB, 115 DAB, mature stage and fruit senescence stage). The transcription levels of the *PbMCs w*ere measured using FPKM values (Fig 6C). Of the 12 *MC* genes identified in *P*. *bretschneideri*, nine *PbMCs* were detected to be expressed in one or more developmental stages, and three genes (*PbMC01*, *PbMC03* and *PbMC04*) out of nine *PbMCs* were identified to be expressed in all seven fruit development stages (Fig 6C). Notably, several *PbMCs* continuously reduced or increased at one or several stages, such as *PbMC01* and *PbMC12*, which were up-regulated in Fruit_stage4 (85 DAB), *PbMC02*, *PbMC06* and *PbMC10*, which were highly expressed at Fruit_stage1 (15 DAB), *PbMC07* was up-regulated in Fruit_stage6 (mature stage), indicating that these gene play an important role in the fruit-specific developmental stages.

## Discussion

In recent studies, the vast majority of published articles suggested that the *MC* genes exist as multi-gene families in the genome [2, 18, 19]. Although the number of *MC* gene family have been investigated in several species [18–23], such as *Arabidopsis thaliana*, *Vitis vinifera*, *Hevea brasiliensis* and *Oryza sativa*. However, the *MC* genes from Rosaceae genomes have not been characterized in detail to our knowledge. In the present study, 70 *MC* genes were identified in six Rosaceae genomes; 12 of these sequences were from *P*. *bretschneideri*, and 8, 7, 8, 12, and 23 *MC* genes in *F*. *vesca*, *P*. *mume*, *P*. *persica*, *P*. *communis* and *M*. *domestica*, *respectively*. By using the MEROPS online tool (https://www.ebi.ac.uk/merops/) [2], we predicted the distribution of Type-I* (8), Type-I (42) and Type-II (20) *MC* genes in these genomes, which consistent with the predictions of phylogenetic analysis of these *MC* gene family numbers. Using a comparative genomic approach, Fagundes et al. (2015) identified *MC* genes in *Viridiplantae*, and then the distribution of Type-I and -II *MC* genes was predicted in 42 plant species [1]. They found that the total number of Type-I *MC* genes was more than twice the number of Type-II *MC* genes [1], which is basically consistent with our results. The diversity of gene structure not only provides additional evidence for supporting phylogenetic groupings, but it also plays an important role in the evolution of gene families [58]. Exon-intron structure analysis suggested that the most of Type I *MC* genes contained four or five exons and Type II *MC* genes had two or three exons. We also noted that the lengths of the exons were more conserved than the introns in Type I *MC* genes (Fig 2). Our data was consistent with the previous published articles that Type I *MC* genes contained more exon numbers than Type II *MC* genes [1, 22]. Additionally, the identification and phylogenetic classification of *MC* genes were further supported by the exon-intron structure analysis in Rosaceae genomes.

Gene duplication plays an important role in the process of biological evolution [59]. In some plants, microsynteny has been extensively described, such as *GELP*, *WOX* and *MYB* gene family in Rosaceae genomes [39, 41, 46]. In the present study, based on the microsynteny analysis, we did not observe the microsynteny relationships among *FvMC01*, *FvMC03*, *FvMC04*, *FvMC06* and *FvMC07*, *PcMC09*, *PcMC10*, *PcMC11* and *PcMC12*, and 22 with other *MC* genes in these four species (*P*. *mume*, *P*. *persica*, *P*. *bretschneideri* and *M*. *domestica*), implying that either these genes were formed through complete transposition and loss of their primogenitors or they were ancient genes without detectable linkage to other *M*C genes. Remarkably, two or more *MC* genes from these Rosaceae genomes (*F*. *vesca*, *P*. *mume*, *P*. *persica*, *P*. *communis*, *P*. *bretschneideri* and *M*. *domestica*) were orthologous to the *MC* genes of the same species, indicating that these gene pairs may play important roles in the expansion of *MC* gene family during evolution, such as *PcMC0*1, *PcMC04* and *PcMC05* are orthologous genes to *PbMC03*, as well as *PbMC04* and *PbMC09* are orthologous genes to *PcMC08*.

So far, there is increasing evidence that MC activity plays an important role for PCD in plant growth and development, and it is essential for plant pollen and embryonic development [1–4, 60]. For example, Watanabe et al. (2011) were observed the stronger GUS expression of *AtMCP2d* promoter in vascular bundles of roots, pollen and embryonic cells [61]. In the present study, we explored the expression pattern of *MC* genes in pear pollen tube development and fruit development. Previous studies have shown that different abiotic stresses can induce PCD, and abiotic stress-induced PCD has a significant effect on plant growth and development [18–22, 62]. *MC* genes have also been shown to play key roles in the abiotic stress response in plants. *Oryza sativa MC* genes showed differential expression patterns in response to cold, drought, and salt stresses, and the expression of most *OsMC* genes was down-regulated when responding to salt and drought stresses [63]. In *Hevea brasiliensis*, Liu et al. (2016) reported that all of the *HbMCs*, except *HbMC5*, displayed transcriptional changes when responding to salt, drought, as well as cold stresses [19]. In *Hordeum vulgare*, qPCR analysis suggested that the expression of *HvMC4* was significantly increased upon excess-B supplementation [23]. In the present study, we found that several *PbMC* genes also respond to drought stresses, including *PbMC03*, *PbMC04* and *PbMC08*. Interestingly, the *PbMC08* shown continuous down-regulated after a long period of stress treatment. In contrast, the *PbMC04* gene was strongly and rapidly induced by drought treatment. The opposite expression pattern indicates that different *PbMC* genes contained divergent regulatory mechanisms to respond drought stress. Our data suggested that *PbMC* genes might play an important role in pear reproductive development and abiotic response.

## Conclusion

In the present study, 70 *MC* genes were identified in Rosaceae genomes, which including 8, 7, 8, 12, 12, and 23 *MC* genes in the genomes of *F*. *vesca*, *P*. *mume*, *P*. *persica*, *P*. *communis*, *P*. *bretschneideri* and *M*. *domestica*, respectively. Subsequently, we carried out comparative genomic and systematic analysis, such as phylogenetic relationships, exon-intron structures, microsynteny, conserved motifs, and expression patterns. Our results suggested the vast majority of *MC* gene of *P*. *communis*, *P*. *bretschneideri* and *M*. *domestica* was expanded by large-scale gene duplication. Expression profiling revealed that *PbMC01* and *PbMC03* were able to be detected in all four pear pollen tube and seven fruit development stages. This understanding of *MC* expression provides a new avenue for functional analyses of pear during pollen tube and fruit development.

## Supporting information

S1 Fig Phylogenetic tree of *MC* genes from six Rosaceae species (*F*. *vesca*, *P*. *mume*, *P*. *persica*, *P*. *communis*, *P*. *bretschneideri* and *M*. *domestica*) and *Arabidopsis thaliana*. The Maximum-likelihood tree was generated using IQ-TREE software, and *MsMC* (CBN76943.1) from *Ectocarpus siliculosus* was used as the out-group.
(PDF)

S1 Table Primers in this study. (XLSX)

S2 Table Information of *MC* genes in six Rosaceae species, including *F*. *vesca*, *P*. *mume*, *P*. *persica*, *P*. *communis*, *P*. *bretschneideri* and *M*. *domestica*. (XLSX)

S3 Table Twenty MEME motif sequences of MC proteins in six Rosaceae species. (XLSX)

S4 Table Homologous gene pairs of MC among between *F*. *vesca*, *P*. *mume*, *P*. *persica*, *P*. *communis*, *P*. *bretschneideri* and *M*. *domestica*. (XLSX)
