# Supplementary figures and images for: Metacaspase gene family in Rosaceae genomes: Comparative genomic analysis and their expression during pear pollen tube and fruit development
Source: PLoS One. 2019 Feb 22;14(2):e0211635. doi: 10.1371/journal.pone.0211635 (PMC6386261; doi:10.1371/journal.pone.0211635)

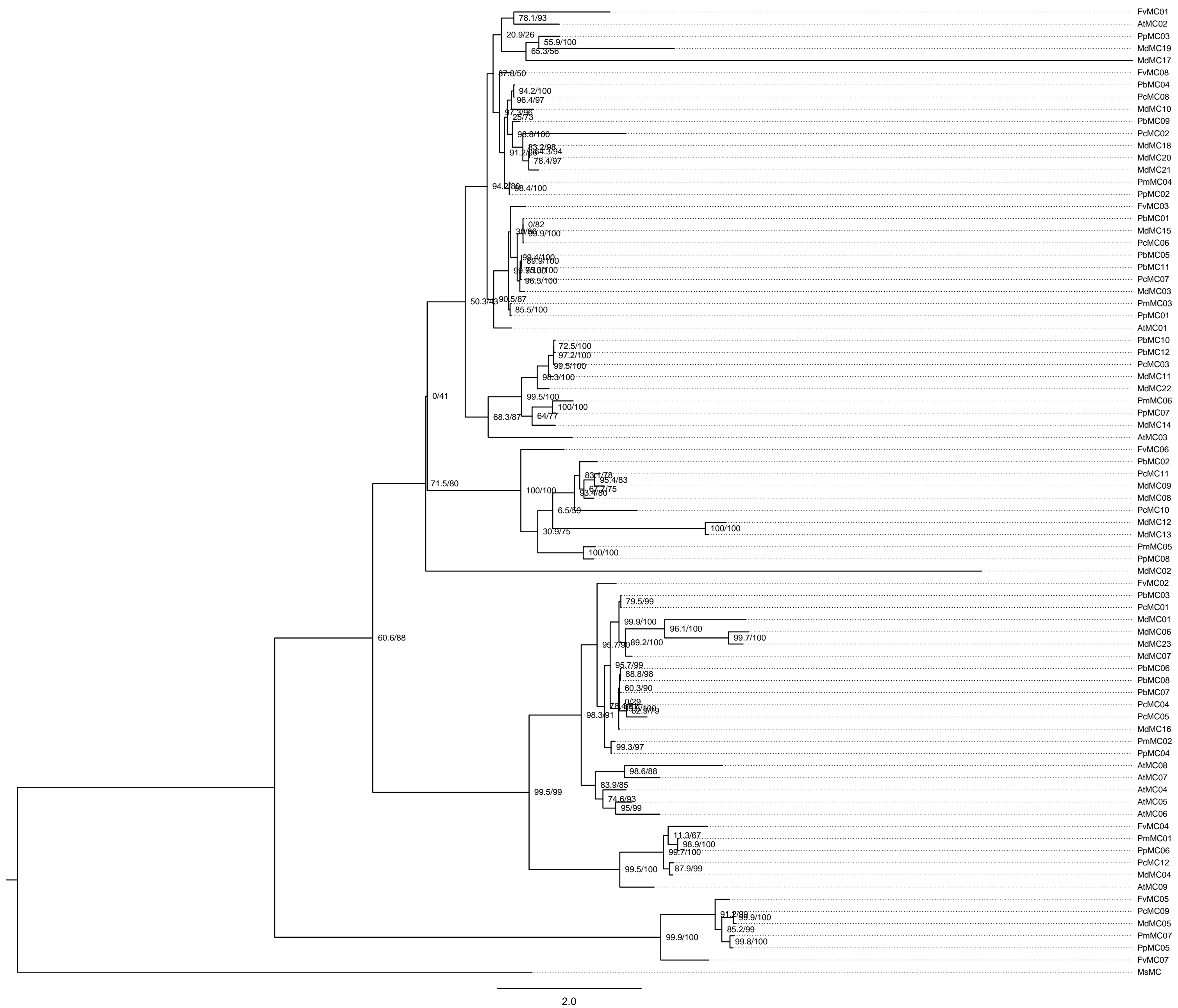

Supplement: S1 Fig — The Maximum-likelihood tree was generated using IQ-TREE software, and MsMC (CBN76943.1) from Ectocarpus siliculosus was used as the out-group. (PDF) [file pone.0211635.s001.pdf]
